# Seroprevalence and risk factors for *Toxoplasma gondii* in sheep in Grosseto district, Tuscany, Italy

**DOI:** 10.1186/1746-6148-9-25

**Published:** 2013-02-07

**Authors:** Beniamino T Cenci-Goga, Antonio Ciampelli, Paola Sechi, Fabrizia Veronesi, Iolanda Moretta, Valentina Cambiotti, Peter N Thompson

**Affiliations:** 1 Dipartimento di Medicina Veterinaria, Università degli Studi di Perugia, Via San Costanzo, 06121, Perugia, Italy; 2 Veterinary practitioner, Tuscany, Italy; 3 Epidemiology Section, Department of Production Animal Studies, Faculty of Veterinary Science, University of Pretoria, Private Bag X04, Onderstepoort, 0110, South Africa

**Keywords:** Toxoplasmosis, Sheep, Prevalence, Intra-cluster correlation coefficient, IFAT, Risk factors

## Abstract

**Background:**

Serum samples from 630 milk sheep, in 33 dairy flocks representative of the southern area of the Tuscany region, were tested for the presence of antibodies to *Toxoplasma gondii* using an indirect immunofluorescence antibody test (IFAT). Questionnaires exploring the management system were completed by the veterinarian in charge of the flocks.

**Results:**

At least one seropositive animal was found in 32 of the 33 flocks tested (97.0%; 95% CI: 84.2%, 99.9%). In the positive flocks, median seroprevalence was 29.4% (interquartile range: 15.9%-46.1%). Overall animal-level seroprevalence, adjusted for sampling weights and test sensitivity and specificity, was 33.3% (95% CI: 24.8%, 42.7%). In a multivariable negative binomial regression model the number of seropositive animals in a flock decreased with increasing flock size (for >400 vs. <300 animals: count ratio (*CR*) = 0.62; 95% CI: 0.41, 0.95; *P* = 0.028) and was greater on farms where stray cats had access to animals’ water (*CR* = 1.54; 95% CI: 1.05, 2.26; *P* = 0.027).

**Conclusions:**

Small flock size and access of cats to water are potential risk factors for *Toxoplasma* infection in sheep in the Grosseto district in Tuscany, Italy. Sheep could be an important source of *T*. *gondii* infection in humans, since we estimate that between 25% and 43% of sheep in the district were seropositive. Toxoplasmosis is also likely to be an important cause of abortion in sheep in the district. Control and prophylactic measures must be adopted to improve the rearing system and the implementation of health promoting programmes in a joint effort between sheep farmers, farmers’ associations and veterinarians to inform about the means of transmission of the infection and for a better understanding of the disease.

## Background

*Toxoplasma gondii* is an intracellular protozoan parasite with felids as definitive hosts and a wide range of warm-blooded animals, including humans and domestic animals, as intermediate hosts [1-3]. Amongst food producing animals, sheep are the most susceptible to infection and *T*. *gondii* is an important cause of abortions and stillbirths [1]. Toxoplasmosis is a source of economic loss for sheep farmers and a danger for consumers, who can be infected by undercooked meat and untreated milk, with severe consequences mostly for pregnant women and immunocompromised individuals [2,4,5].

Sheep and goats are an important source of infected meat in southern European countries [6,7]. Tachyzoites of *T*. *gondii* have also been detected in the milk of several intermediate hosts, including sheep, goats and cows [7]. Although the results of a recent study [8] demonstrate that, even with a low prevalence of infection in cattle, consumption of beef remains an important source of infection, lamb meat is an important risk factor, as determined by many case-control studies [9]. Seropositive sheep can be assumed to harbour large numbers of tissue cysts in their meat [10]. Sheep and lambs are usually kept on pasture and are therefore at risk of infection due to contamination of the environment with sporulated oocysts. If the environment is heavily contaminated with oocysts, infection prevalence can exceed 90% [5,11]. This is of particular importance because tissue cysts have been found in many edible parts of sheep [12] and small ruminants are an important source of proteins (milk and meat) both in industrialized and developing countries. Considering the fact that in some countries raw or under-cooked lamb is considered an elite food, we can conclude that consumption of under-cooked lamb is an important risk factor for toxoplasmosis in humans. In countries where mutton is eaten well-cooked, the risk is much reduced [1].

Several studies have been conducted to establish the seroprevalence of *T*. *gondii* in sheep in different countries. Results range from 32.9% [13] to 80% [14] in Brazil, 84.5% in Serbia [15], 52.2% in Saudi Arabia [16], 29.1% in Iran [17], and 5.6% in South Africa [11]. In southern Italy, a cross-sectional study conducted in the Campania region found a seroprevalence of 28.5% in pastured sheep [18]. In addition, a study performed during the period 1999-2002 showed a seroprevalence of 28.4% in sheep in Sardinia, Italy [19]. Differences in seroprevalence between countries and regions may be due to different climatic and other risk factors as well as to differing cut-off values used to define seropositivity to the serological test [1,20,21]. Risk factors associated with infection are mainly related to animals (age, sex), rearing system, presence of cats on the farm, and farm management [11,13,15,19,22].

In an attempt to quantify the prevalence of *T*. *gondii* infection and further understand the potential risk posed to humans in central Italy, a study was undertaken to determine seroprevalence and risk factors for T. *gondii* infection in sheep herds in Tuscany. This is the first time that such a study has been done in the region, therefore no data are available in literature.

## Methods

The research protocol for this study was approved by the council of the Dipartimento di Scienze Biopatologiche, no. 5, 28.09.2009

### Study population and sample size

**Figure 1:**
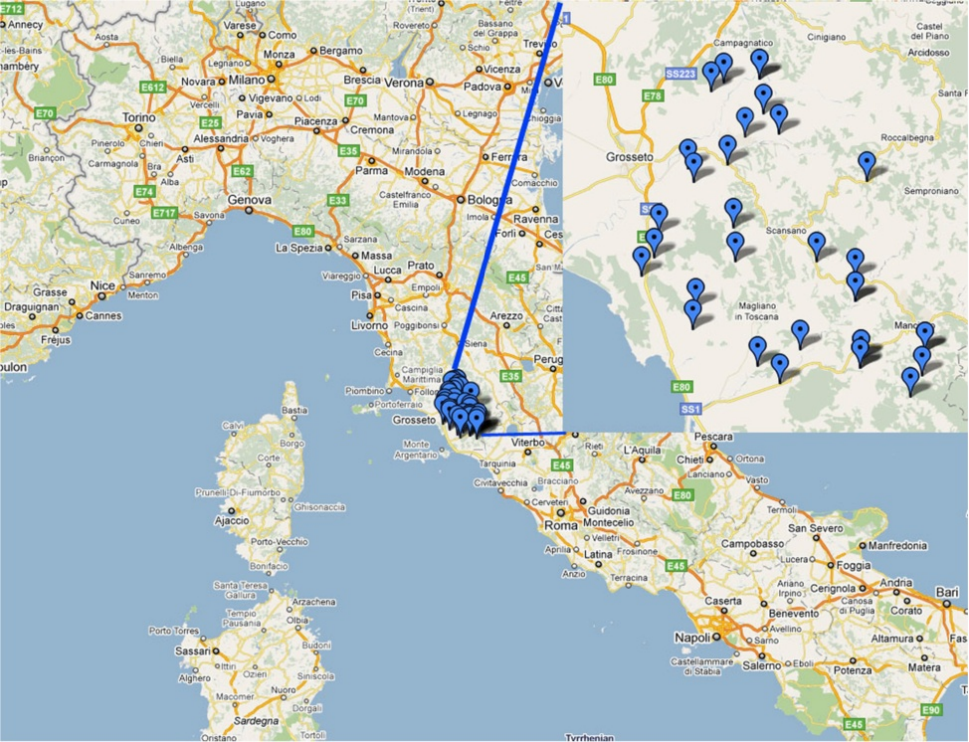
Study area and sampled farms.

Serum samples were collected from adult sheep (>18 months old) of the Sarda breed in dairy flocks in Grosseto district, Tuscany, Italy during May-June 2011. The study area extends from latitude 42.851078°N to 42.51902°N and from longitude 11.129635°E to 11.531657°E (Figure 1). The area is mainly hilly and the altitude of sampled farms ranged from 13 to 574 m above sea level. Sheep farming in Tuscany is very popular and the region has the fourth highest sheep population in Italy, the three highest being the islands of Sardinia and Sicily, and neighbouring Lazio [23]. Grosseto district has approximately 730 sheep flocks and 122,000 sheep. None of the animals had ever been vaccinated against toxoplasmosis; indeed, such vaccination is not practiced in Italy. 

Sample size was calculated using a 25% expected animal-level prevalence, 7.5% absolute error and 95% confidence level. This was adjusted for multi-stage sampling, in which an average of 20 animals per flock would be tested, using a conservative value for the intracluster correlation coefficient (ICC) of *ρ* = 0.20 [24], which gave a required sample size of 620 animals in 31 flocks. This would also allow an estimate of flock-level seroprevalence of approximately 90% with 10% allowable error and 95% confidence. The flocks were selected using simple random sampling from a list of premise-identification numbers to ensure a representative sample of farms from the district. Within each selected flock, adult sheep (>18 months) were sampled by systematic random selection. Between 8 and 45 animals were selected per flock, approximately proportional to the total number of adult sheep present in the flock.

### Questionnaire data collection

A questionnaire (Additional file 1 Audit form on rearing practices from 33 farms in Grosseto district, Tuscany, Italy.) was prepared to include questions on: rearing system (extensive: daily grazing in favourable weather conditions and returning to fold at night or daily grazing with possibility of shelter in bad weather; intensive: sheep housed day and night), water source (stagnant water source, e.g. pond or dam, used for sheep), separate water troughs and separate feeding troughs for young and adult animals, purchase of spare breeding animals, presence of resident and/or stray cats on the property, and access of cats to feed stores and water sources. The questionnaire was administered by the attending veterinarian at the time of blood sample collection and the veterinary practitioner, a co-author of this study, was always present during the interview.

### Serology

Venous blood samples were collected from each selected animal and transferred to the laboratory on ice. After centrifugation (3000 rpm for 10 minutes), the sera were stored at -20°C until analysis [3]. Serum samples were analysed using a commercial indirect immunofluorescence antibody assay (IFAT) to determine the presence of IgG antibodies against *T*. *gondii*. The serological test was performed according to the method of Camargo [25] using slides spotted with whole RH strain tachyzoites (Mega Cor Diagnostic, Horbranz, Austria) as antigens and fluorescein isothiocyanate-labelled rabbit anti-sheep IgG (whole molecule, Sigma-Aldrich, St Luis, MO, USA), diluted 1:100 in PBS plus 0.01% Evans blue, as conjugate. Sera were screened at 1:64 dilution (cut-off) and those testing positive were serially two-fold diluted to determine the end-point titre (the highest dilution of serum that gave a positive reaction). Positive and negative controls were included in each assay and the slides were examined under a fluorescence microscope (Olympus U-RFL-T) at 400 or 1000× magnification. Only a bright, linear, peripheral fluorescence of the tachyzoites was considered positive. Although the manufacturer claimed *Se* and *Sp* both to be >99%, we could not find independent confirmation of this, so for adjustment of apparent prevalence (see below) we used *Se* = 80.4% and *Sp* = 91.4%, reported elsewhere for another commercial IFAT [26].

### Statistical analysis

Within-flock seroprevalence for each farm, flock-level seroprevalence and exact binomial 95% confidence limits were calculated. The ICC (*ρ*), indicating the degree of clustering of seropositivity within flocks, was estimated by using a logistic model of individual animal test outcome, with a random effect for flock and no fixed effects, to obtain the flock random effect variance, *σ*_*f*_^2^, and then calculating *ρ* = *σ*_*f*_^2^ / (*π*^2^/3 + *σ*_*f*_^2^), where *π*^2^/3 is the variance of the logistic distribution [27]. Within each flock, sampling fraction was calculated as the proportion of animals >18 months that was sampled, and sampling weight was calculated as the inverse of the sampling fraction. Using the ‘*svy*’ commands in Stata 12, overall animal-level seroprevalence was calculated by weighting each individual’s result by its sampling weight and the standard error was adjusted for clustering and weighting. Finally, animal-level prevalence estimates and confidence limits were adjusted for test sensitivity (80.4%) and specificity (91.4%) using the formula of Rogan and Gladen [28]: *TP* = (*AP* + *Sp*–1) / (*Se* + *Sp*–1), where *TP* = true prevalence, *AP* = apparent prevalence (after adjusting for sampling weight), *Se* = test sensitivity and *Sp* = test specificity.

The bivariable association between each potential risk factor and within-flock seroprevalence was assessed using a Poisson regression model of the number of seropositive animals in a herd with the number of animals tested as an exposure variable, i.e. *ln*(number tested) was included in the model with its coefficient constrained to 1. Variables associated with the outcome (*P* < 0.20) were then entered into a multivariable Poisson model, with number tested as exposure, which was developed by backward elimination until all remaining variables were significant (*P* < 0.05). All other variables were then re-tested in the model and retained if significant. Fit of the Poisson model was assessed using the Pearson goodness-of-fit test and dispersion parameter [27]. Finally, the corresponding negative binomial regression model of the number of seropositive animals in a herd was compared with the Poisson model using a likelihood ratio test. All analyses were done using Stata 12.1 (StataCorp, College Station, TX, U.S.A.).

## Results

Serum samples were collected from 630 sheep in 33 flocks and tested for antibodies to *T*. *gondii* by IFAT. Flock sizes ranged from 70 to 630 adult sheep (mean: 247; median: 210) and between 8 and 45 animals (mean: 19.1; median: 17) were tested per flock. Sampling fractions within flocks ranged from 0.059 to 0.135 (mean: 0.079; median: 0.075). Using a cut-off titre of 1:64, a total of 214/630 (34.0%) samples tested positive. Among the 214 reactive samples, 42 (19.6%) had a titre of 1:64, 36 (16.8%) of 1:128, 39 (18.2%) of 1:256, and 97 (45.3%) of 1:512 or above.

Overall animal-level seroprevalence in the district, adjusted for sampling weights and for test sensitivity and specificity, was 33.3% (95% CI: 24.8%, 42.7%). At least one seropositive animal was found in 32 of the 33 flocks tested, therefore the flock-level seroprevalence was estimated to be 97.0% (95% CI: 84.2%, 99.9%). In the positive flocks, median seroprevalence was 29.4% (interquartile range: 15.9%, 46.1%). The ICC (*ρ*) was estimated to be 0.211 (95% CI: 0.113, 0.360).

In the bivariable analysis of potential risk factors (Table 1), five variables (flock size, production system, stagnant water, resident cats, access of stray cats to water) were selected for inclusion in the multivariable Poisson model. The final Poisson model contained only two predictors (flock size and access of cats to water) but showed lack of fit (Pearson *χ*^2^_29 *df*_ = 48.0; *P* = 0.015) and evidence of overdispersion (dispersion parameter = 1.65, where >1 indicates overdispersion). Further, the likelihood ratio test comparing the corresponding negative binomial regression model with the Poisson model was marginally significant (*P*_*LRT*_ = 0.052), therefore the negative binomial model was preferred. After re-testing each independent variable for significance, the final negative binomial model is shown in Table 2. Adjusted for the number tested, the number of seropositive animals in the flock decreased with increasing flock size (for >400 vs. <300 animals: count ratio (*CR*) = 0.62; 95% CI: 0.41, 0.95; *P* = 0.028). In addition, the number of seropositive animals was greater on farms where stray cats had access to animals’ water (*CR* = 1.54; 95% CI: 1.05, 2.26; *P* = 0.027).

**Table 1:** **Mean within-farm seroprevalence of *Toxoplasma gondii *in dairy sheep flocks and bivariable association with potential risk factors**

**Variable**	**Level**	**Number of farms**	**Mean seroprevalence(%)**	***P***-value^*^
Size of flock	<300	14	42.4	<0.001
	300-400	10	33.5	
	>400	9	24.0	
Production system	Extensive	30	35.9	0.088
	Intensive	3	22.6	
Stagnant water used	No	21	31.3	0.05
	Yes	12	40.7	
Separate water trough for young and adult animals	No	29	33.7	0.587
	Yes	4	42.2	
Separate feed troughs for young and adult animals	No	10	31.3	0.948
	Yes	23	36.18	
Purchased breeding animals in last 5 years	No	3	22.7	0.481
	Yes	30	35.9	
Resident cats present on farm	No	10	28.4	0.122
	Yes	23	37.4	
Stray cats occur on farm	No	0	–	–
	Yes	33	34.7	
Access of stray cats to animal feed	No	7	32.4	0.715
	Yes	26	35.3	
Access of stray cats to animals’ water	No	12	22.9	0.001
	Yes	21	41.4	

^*^ From Poisson regression model with number tested as offset.

**Table 2:** **Farm-level factors associated with number of animals seropositive to *Toxoplasma gondii *in 33 dairy sheep flocks: results of a negative binomial regression model**

**Variable**	**Level**	**Count ratio**	**95%CI(CR)**	***P-*value**
Size of flock	<300	1^*^	–	–
	300-400	0.83	0.55, 1.26	0.377
	>400	0.62	0.41, 0.95	0.028
Access of stray cats to animals’ water	No	1^*^	–	–
	Yes	1.54	1.05, 2.26	0.027
*ln*(number tested)	(exposure)	–	–	–

^*^ Reference level.

## Discussion

There has been an increasing interest in recent years in the prevalence of *T*. *gondii* infection in small ruminants because of their role on the dissemination of the protozoan to man through direct contact or by consuming products of animal origin [1,11,14,22,29-31]. Although the average seroprevalence of *T*. *gondii* infection in sheep populations has long been estimated to be approximately 30% [32], very few studies have been conducted in Italy and little is known about the prevalence of *T*. *gondii* in many areas where sheep farming is an important element of the local economy. Lamb, mutton and sheep milk are important sources of human *T*. *gondii* infections, but actual data on the prevalence of *T*. *gondii* in sheep are scarce [1]. This study estimated the seroprevalence of *T*. *gondii* in dairy sheep in Tuscany, Italy, and investigated risk factors associated with the number of infected animals in a flock. The test of choice was a commercial indirect fluorescent antibody test (IFAT). Although the variability of the sensitivity and specificity depending on the *Toxoplasma* strain employed, and the subjectivity in interpreting the fluorescence reaction make it difficult to compare results between laboratories, the IFAT is simple to carry out and can easily be performed for large numbers of samples and several dilutions, even by inexpert lab technicians [1]. Data in the literature show some variability in sensitivity and specificity even in the same regions and in the same type of farm [26,33,34]. A recent study [33] has revealed that there is poor agreement between IFAT and the modified agglutination test (MAT) in cat and dog sera. Moreover most surveys are performed on blood collected at the slaughterhouse after sticking, resulting in mixing of arterial and venous blood. The latter is required by good laboratory practice for serology, whereas the different pH and composition of arterial blood may influence the test sensitivity and specificity [35]. For our study we could not obtain reliable estimates of test sensitivity or specificity for the particular IFAT used, and therefore chose to use more conservative estimates from a different IFAT. However, with the high prevalence encountered in the study, the adjustment for test performance made very little change to the estimate and therefore did not materially influence our results.

In the present study, a 33.3% seroprevalence (95% CI: 24.8%, 42.7%) of *T*. *gondii* infection in sheep raised in the Grosseto district, Tuscany, Italy, was detected. When using multistage or cluster sampling, the standard error (SE) of the prevalence estimate is increased compared with simple random sampling of the same number of animals [8], and this should be taken into account when calculating a confidence interval for the prevalence estimate. In addition, when sampling fractions differ between clusters this should be adjusted for in calculating the point estimate of the prevalence. Ignoring the above adjustments, as well as the adjustment for test sensitivity and specificity, would have resulted in a seroprevalence estimate of 34.0% (95% CI: 30.3%, 37.8%). Our estimate of 33.3% (95% CI: 24.8%, 42.7%) therefore provides a more realistic measure of the precision of the study. The increase in SE with multi-stage sampling depends on the average number of units sampled from each cluster, as well as the ICC. The estimated ICC of *ρ* = 0.211 indicates moderate clustering of infection within flocks and should be taken into account when calculating sample sizes for future surveys.

Comparison of our results with those of previous studies should be done with care since various assays have been used. Over a one year period following 31 abortions caused by *T*. *gondii* on a typical farm of 524 sheep in Italy, the prevalence of IgG positives ranged from 31.5% at the first sampling to 62.6% at the fourth sampling [36]. Authors [37] reported an overall seroprevalence of 51.3% in Sardinia using an ELISA test; seroprevalence increased significantly with age. In a cross-sectional serological survey to evaluate, irrespective of abortion, the *T*. *gondii* infection in pastured sheep from the Campania region of Southern Italy, 77.8% of the farms and 28.5% of the sheep tested positive [18]. In addition, the presence of *T*. *gondii* DNA was detected by PCR in 4/117 milk samples (3.4%). Analysis of sera from 1961 sheep, collected just before slaughtering from 62 farms in Sicily revealed a seroprevalence of 49.9% using a commercially available ELISA for *Toxoplasma*-specific IgG. Eighty-seven percent of the farms had at least one seropositive animal [38].

The worldwide seroprevalence of *T*. *gondii* infection in sheep was recently reviewed by Dubey [2]. Since then, seroprevalences of 44.1% have been reported in sheep from Grenada and Carriacou, West-Indies [39] and 18.6% in Sao Paulo, Brazil [40], using MAT. In a serological survey of *T*. *gondii* infection in adult breeding sheep in Great Britain, of the 3539 sera collected from 227 flocks, 74% were found to be positive for *T*. *gondii* specific antibody using latex agglutination [41]. Multilevel logistic modelling suggested that the likelihood of infection increased with age and this effect appeared to be amplified in animals vaccinated against *T*. *gondii*. The model also indicated that the odds of sheep being seropositive were increased on premises where cattle were also kept. These results suggest a high level of *Toxoplasma* infection in breeding sheep in Great Britain and provide further evidence to suggest that postnatal infection is more common than congenital infection in sheep.

The seroprevalence of more than 30% demonstrated in this study is therefore consistent with several previous reports [18,19,36], but lower than that reported by others [37-39,41]. The variability of these results may be due to the differences in the age and management of the sampled animals, the environment and in the serological technique used [1,34].

In the multivariable model, a strong positive association was found between within-farm seroprevalence of *T*. *gondii* and the access of cats to water given to animals, confirming that contact with feline species is important in the epidemiology of toxoplasmosis. The fact that cats, either resident or stray, were present on every farm could explain the high prevalence of *T*. *gondii*-specific antibodies observed in sheep in this study, due to elimination of oocysts by the cats and contamination of the environment. The strong association between seroprevalence and access of cats to water used by the sheep, but no association with access of cats to the feed store, suggests that contaminated water may be as important, if not more important a source of infection than contaminated feed. This supports the conclusion of a recent review paper [24] that waterborne transmission of *T*. *gondii* may be more common than previously thought. In a previous European study, the “use of surface water sources for drinking” was identified as a putative risk factor for *T*. *gondii* seropositivity in sheep [38].

In the initial bivariable analysis, but not in the multivariable analysis, there was an association between seroprevalence and the use of stagnant water, which may have been due to the fact that cats were more likely to have had access to stagnant water than to running water. As oocyst survival in soil for up to two years has been reported, any faecal material from infected cats will represent a hazard [1]. *T*. *gondii* oocysts survive in water (up to 54 months in cold water) so unfiltered water contaminated with *T*. *gondii* can lead to infection [42,43].

After adjusting for the number of sheep tested in the flock, as well as for confounding due to the other risk factors assessed, the number of seropositive animals in the flock was negatively associated with the size of the flock; seroprevalence was therefore higher in smaller flocks. Since all but three of the flocks in our study were classified as having an extensive management system, the association of this variable with seroprevalence could not be assessed. However, in our study area the farms with higher numbers of sheep tended to be more intensive, therefore the negative association between seroprevalence and flock size may have been due to unmeasured management or environmental factors related to degree of intensification. This would be consistent with the report of WNA van der Puije, et al. [44] that prevalence was higher in extensively managed sheep flocks. Possible reasons could be that some smaller, more extensively managed sheep flocks may have had more exposure to cats in the environment, or to contaminated stagnant pools (apart from their primary water source noted in the questionnaire). On the other hand, a recent sero-epidemiological study conducted in Greece [27] showed infection rates to be significantly higher in intensive rearing systems due to the higher exposure of animals to contaminated feed. In addition, farm facilities under intensive or semi-intensive conditions may provide shelter to various hosts of *T*. *gondii* (such as cats and rodents) which might be involved in the spread of infection [27].

No association with seropositivity was observed for the use of separate water and feed troughs for young and adult animals or for the purchase of spare breeding animals, supporting the prevailing view that direct sheep to sheep spread at lambing has not been proven and that transmission from rams at mating has been dismissed as a route of infection [2].

## Conclusion

Based on the results obtained in this study, it can be concluded that small flock size and access of cats to drinking water are potential risk factors for *Toxoplasma* infection in sheep in the Grosseto district in Tuscany, Italy. Sheep could be an important source of *T*. *gondii* infection in humans, since we estimate that between 25% and 43% of sheep in the district are seropositive. Toxoplasmosis is also likely to be an important cause of abortion in sheep in the district. Control and prophylactic measures must be adopted to improve the rearing system and the implementation of health promoting programmes in a joint effort between sheep farmers, farmers’ associations and veterinarians to inform about the means of transmission of the infection and for a better understanding of the disease.

## Competing interests

The authors declare that they have no competing interests.

## Authors’ contributions

The study idea was conceived by BCG. BCG, PS and AC participated in the design of the study. FV and IM participated in the acquisition of the laboratory data. AC collected serum samples with the attending veterinarians and helped administering the questionnaire. PT and VC provided previously acquired reference data. BCG and PT carried out the statistical analysis. Data interpretation was done by all authors. BCG and PT drafted the manuscript. All authors contributed to the critical revision of the manuscript for important intellectual content and have seen and approved the final draft.

## Supplementary Material

Additional file 1 Audit form on rearing practices from 33 farms in Grosseto district, Tuscany, Italy. Click here for file

## References

[B1] Cenci Goga BT Rossitto PV Sechi P Mc Crindle CME Cullor JS Toxoplasma in animals, food and humans: an old parasite of new concern Foodborne pathog dis 2011 Vol. 8- n.7 1 12 10.1089/fpd.2010.0795 21486145

[B2] Dubey JP Toxoplasmosis in sheep - The last 20 years Vet Parasitol 2009 163 1-2 1 14 10.1016/j.vetpar.2009.02.026 19395175

[B3] Urquart GM Armour J Duncan JL Dunn AM Jennings FW Parassitologia veterinaria 2005 2 UTET, Milano, Italy

[B4] Innes EA Bartley PM Buxton D Katzer F Ovine toxoplasmosis Parasitology 2009 136 14 1887 1894 10.1017/S0031182009991636 19995468

[B5] Tenter AM Heckeroth A Weiss LM *Toxoplasma gondi*, from animals to humans Int J Parasitol 2000 30 12–13 1217 1258 11113252 10.1016/s0020-7519(00)00124-7 PMC3109627

[B6] Clancy R Ren Z Turton J Pang G Wettstein A Molecular evidence for Mycobacterium avium subspecies paratuberculosis (MAP) in Crohn’s disease correlates with enhanced TNF-alpha secretion Digestive Liver Dis 2007 39 445 451 5, May, 2007 10.1016/j.dld.2006.12.006 17317344

[B7] Tenter AM *Toxoplasma gondii* in animals used for human consumption Memorias do Instituto Oswaldo Cruz 2009 104 364 369 10.1590/S0074-02762009000200033 19430665

[B8] Opsteegh M Prickaerts S Frankena K Evers EG A quantitative microbial risk assessment for meatborne Toxoplasma gondii infection in The Netherlands Int J Food Microbiol 2011 150 2–3 103 114 21864927 10.1016/j.ijfoodmicro.2011.07.022

[B9] Anonymous Toxoplasmose: état des connaisances et évaluation du risque lié à l’alimentation In AFSSA - Report of the Afssa working group “Toxoplasma gondii”, University of Pretoria. 2005 http://www.afssa.fr/Documents/MIC-Ra-Toxoplasmose.pdf

[B10] Dubey JP Jones JL *Toxoplasma gondii* infection in humans and animals in the United States Int J Parasitol 2008 38 11 1257 1278 10.1016/j.ijpara.2008.03.007 18508057

[B11] Samra NA Mc Crindle CM Penzhorn BL Cenci Goga BT Seroprevalence of toxoplasmosis in sheep in South Africa JS AfrVet Assoc 2007 78 3 116 120 10.4102/jsava.v78i3.301 18237032

[B12] Dubey JP Kirkbride CA Enzootic toxoplasmosis in sheep in North-Central United-States J Parasitol 1989 75 5 673 676 10.2307/3283047 2795369

[B13] Pinheiro JJW Mota RA da Fonseca Oliveira AA Faria EB Gondim LFP da Silva AV Anderlini GA Prevalence and risk factors associated to infection by *Toxoplasma gondii* in ovine in the state of alagoas, Brazil Parasitol Res 2009 105 709 715 10.1007/s00436-009-1472-3 19468755

[B14] Rossi GF Cabral DD Ribeiro DP Pajuaba AC Correa RR Moreira RQ Mineo TW Mineo JR Silva DA Evaluation of *Toxoplasma gondii* and *neospora caninum* infections in sheep from Uberlandia, minas gerais state, Brazil, by different serological methods Vet Parasitol 2011 175 3–4 252 259 21075529 10.1016/j.vetpar.2010.10.017

[B15] Klun I Djurkovic O Katic-Radivojevic S Nikolic A Cross-sectional survey on Toxoplasma gondii infection in cattle, sheep and pigs in Serbia: seroprevalence and risk factors Vet Parasitol 2006 135 121 131 10.1016/j.vetpar.2005.08.010 16188388

[B16] Sanad MM Al Ghabban AJ Serological survey on toxoplasmosis among slaughtered sheep and goats in Tabouk, Saudi Arabia J Egypt Soc Parasitol 2007 37 1 329 340 17580587

[B17] Bonyadian M Hematzade F Manuchehri K Seroprevalence of antibodies to *Toxoplasma gondii* in sheep in center of Iran Pak J Biol Sci 2007 10 18 3228 3230 19090132 10.3923/pjbs.2007.3228.3230

[B18] Fusco G Rinaldi L Guarino A Proroga YT Pesce A de Giuseppina M Cringoli G Toxoplasma gondii in sheep from the Campania region (Italy) Vet Parasitol 2007 149 3-4 271 274 10.1016/j.vetpar.2007.07.020 17764846

[B19] Masala G Porcu R Madau L Tanda A Ilba B Satta G Tola S Survey of ovine and caprine toxoplasmosis by IFAT and PCR assays in Sardinia, Italy Vet Parasitol 2003 117 1-2 15 21 10.1016/j.vetpar.2003.07.012 14597274

[B20] Cenci Goga BT Toxoplasmosi negli animali, negli alimenti e nell’uomo. Una sfida per il medico veterinario – prima parte Praxis veterinaria 2009 Vol. xxx- n.3 7 15

[B21] Cenci Goga BT Toxoplasmosi negli animali, negli alimenti e nell’uomo. Una sfida per il medico veterinario – seconda parte Praxis veterinaria 2009 Vol. xxx- n.4 7 12

[B22] Lopes WD Santos TR da Silva Rdos S Rossanese WM de Souza FA de Faria Rogrigues JD de Mendonça RP Soares VE Costa AJ Seroprevalence and risk factors for Toxoplasma gondii in sheep raised in the jabotical microregion, Sao Paulo State, Brazil Res Vet Sci 2010 88 1 104 106 10.1016/j.rvsc.2009.06.006 19589550

[B23] Anonymous Anagrafe nazionale zootecnica - statistiche 2012 http://www.izs.it/IZS

[B24] Otte M Gumm I Intra-cluster correlation coefficients of 20 infections calculated from the results of cluster-sample surveys Prev Vet Med 1997 31 147 150 10.1016/S0167-5877(96)01108-7 9234433

[B25] Camargo ME Introdução às técnicas de imunofluorescência Revista Brasileira de Patologia Clí nica 1974 10 143 171

[B26] Shaapan RM El-Nawawi FA Tawfik MAA Sensitivity and specificity of various serological tests for the detection of *Toxoplasma gondii* infection in naturally infected sheep Vet Parasitol 2008 153 3–4 359 362 18406534 10.1016/j.vetpar.2008.02.016

[B27] Dohoo IRMW Stryhn H Veterinary epidemiologic research 2009 2 Charlottetown: VER Inc

[B28] Rogan W Gladen B Estimating prevalence from the results of a screening test Am J Epidemiol 1978 107 71 76 623091 10.1093/oxfordjournals.aje.a112510

[B29] Alvarado-Esquivel C García-Machado C Alvarado-Esquivel D Vitela-Corrales J Villena I Dubey JP Seroprevalence of *Toxoplasma gondii* infection in domestic sheep in Durango state, Mexico J Parasitol 2012 98 2 271 273 10.1645/GE-2958.1 21916621

[B30] Opsteegh M Teunis P Mensink M Züchner L Lothar Z Titilincu A Langelaar M van der Giessen J Evaluation of ELISA test characteristics and estimation of Toxoplasma gondii seroprevalence in Dutch sheep using mixture models Prevent Veterinary Med 2010 96 3-4 232 240 10.1016/j.prevetmed.2010.06.009 20637514

[B31] Ueno TE Gonçalves VS Heinemann MB Dilli TL Akimoto BM de Souza SL Gennari SM Soares RM Prevalence of Toxoplasma gondii and neospora caninum infections in sheep from federal district, central region of Brazil Trop Anim Health Prod 2009 41 4 547 552 10.1007/s11250-008-9220-8 18726165

[B32] Blewett DA The epidemiology of ovine toxoplasmosis. I. The interpretation of data for the prevalence of antibody in sheep and other host species Br Vet J 1983 139 537 545 6652460 10.1016/s0007-1935(17)30341-x

[B33] Macrì G Sala M Linder A Pettirossi N Scarpulla M Comparison of indirect fluorescent antibody test and modified agglutination test for detecting *Toxoplasma gondii* immunoglobulin G antibodies in dog and cat Parasitol Res 2009 105 1 35 40 10.1007/s00436-009-1358-4 19221795

[B34] Piergili Fioretti D Problematiche e limiti dei metodi convenzionali ed innovativi nella diagnosi di Toxoplasmosi nell’uomo e negli animali Parassitologia 2004 46 177 181 15305712

[B35] Anonymous Single-use containers for venous blood specimen collection ISO 1995 6710 1995

[B36] Zedda MT Rolesu S Pau S Rosati I Ledda S Satta G Patta C Masala G Epidemiological study of Toxoplasma gondii infection in ovine breeding Zoonoses and Public Health 2010 57 7–8 e102 e108 19968848 10.1111/j.1863-2378.2009.01292.x

[B37] Natale A Porqueddu M Capelli G Mocci G Marras A Sanna Coccone GN Garippa G Scala A Sero-epidemiological update on sheep toxoplasmosis in Sardinia, Italy Parassitologia 2007 49 4 235 238 18689234

[B38] Vesco G Buffolano W La Chiusa S Mancuso G Caracappa S Chianca A Villari S Currò V Liga F Petersen E Toxoplasma gondii infections in sheep in Sicily, southern Italy Vet Parasitol 2007 146 1‚Äì2 3 8 17383099 10.1016/j.vetpar.2007.02.019

[B39] Chikweto A Kumthekar S Tiwari K Nyack B Deokar MS Stratton G Macpherson CNL Sharma RN Dubey JP Seroprevalence of Toxoplasma gondii in pigs, sheep, goats, and cattle from Grenada and carriacou, west Indies J Parasitol 2011 97 5 950 951 10.1645/GE-2811.1 21506801

[B40] Langoni H Greca Júnior H Guimarães FF Ullmann LS Gaio FC Uehara RS Rosa EP Amorim RM Da Silva RC Serological profile of Toxoplasma gondii and neospora caninum infection in commercial sheep from Sào Paulo State, Brazil Vet Parasitol 2011 177 1–2 50 54 21256676 10.1016/j.vetpar.2010.11.024

[B41] Hutchinson JP Wear AR Lambton SL Smith RP Pritchard GC Survey to determine the seroprevalence of Toxoplasma gondii infection in British sheep flocks Vet Rec 2011 169 22 582 10.1136/vr.d5764 21957115

[B42] Dubey JP Beattie CP Toxoplasmosis of animals and Man 1988 CRC Press, Boca Raton, FL, USA

[B43] Dubey JP Toxoplasma gondii oocyst survival under defined temperatures J Parasitol 1998 84 4 862 865 10.2307/3284606 9714227

[B44] van der Puije WNA Bosompem KM Canacoo EA Wastling JM Akanmori BD The prevalence of anti-Toxoplasma gondii antibodies in Ghanaian sheep and goats Acta Trop 2000 76 1 21 26 10.1016/S0001-706X(00)00084-X 10913761

