# Supplementary material for: Seroprevalence and risk factors for Toxoplasma gondii in sheep in Grosseto district, Tuscany, Italy
Source: BMC Vet Res. 2013 Feb 7;9:25. doi: 10.1186/1746-6148-9-25 (PMC3577446; doi:10.1186/1746-6148-9-25)
Supplement: Additional file 1 — Audit form on rearing practices from 33 farms in Grosseto district, Tuscany, Italy. [file 1746-6148-9-25-S1.docx]

Farm: ……………………………………………………………………………………………………..

Number of animals: ………………………………………….

| **Age** | **Number** | | | **Free comments** |
| --- | --- | --- | --- | --- |
| ≤ 12 months |  | | |  |
| between 12 and 18 months |  | | |  |
| ≥ 18 months |  | | |  |
|  |  |  | |  |
| **Production system** | **Yes** | **No** | | **Free comments** |
| Intensive |  |  | |  |
| Semi-extensive |  |  | |  |
| Extensive |  |  | |  |
|  |  |  |  | |
| **Water** | **Yes** | **No** | | **Free comments** |
| Running |  |  | |  |
| Stagnant |  |  | |  |
| Running + stagnant |  |  | |  |
| Separate water trough for young and adult animals |  |  | |  |
|  |  |  |  | |
| **Feeding** | **Yes** | **No** | | **Free comments** |
| Separate feed troughs for young and adult animals |  |  | |  |
|  | | |  | |
| **Reproduction** | **Yes** | **No** | | **Free comments** |
| Purchased breeding animals in last 5 years |  |  | |  |
|  |  |  |  | |
| **Cats** | **Yes** | **No** | | **Free comments** |
| Resident cats present on farm |  |  | |  |
| Stray cats occur on farm |  |  | |  |
| Access of stray cats to animal feed |  |  | |  |
| Access of stray cats to animals’ water |  |  | |  |
